# Anti-Inflammatory Properties of *Garrya flavescens*: Phytochemical Profiling and Mitigation of LPS-Induced Neuroinflammation via ERK Signaling and Mitochondrial Modulation

**DOI:** 10.3390/plants15091319

**Published:** 2026-04-25

**Authors:** Hyun-Jeong Yang, Ohwook Kwon, Dalnim Kim, Ahreum Lee, Kyohee Cho, Hyeran Ihm, Ji Young Kim

**Affiliations:** 1 Department of Integrative Healthcare, University of Brain Education, Cheonan 31228, Republic of Korea; yang@ube.ac.kr (H.-J.Y.); hades770@hanmail.net (O.K.); hoipig0326@gmail.com (D.K.); dkfma5025@gmail.com (A.L.); 2 Korea Institute of Brain Science, Seoul 06022, Republic of Korea; 3 College of Pharmacy, Gachon University, Incheon 21936, Republic of Korea; kcho3138@gmail.com; 4 Department of Integrative Brain Education, Institute of Brain Education, Sedona, AZ 86336, USA; h.ihm@instituteofbraineducation.com; 5 Department of Anesthesiology and Pain Medicine, Yonsei University College of Medicine, Seoul 03722, Republic of Korea

**Keywords:** *Garrya flavescens*, rutin, microglia, LPS, inflammation

## Abstract

*Garrya flavescens S. Wats.* (GF) has been traditionally used to treat gastrointestinal spasms, yet its bioactivity within the central nervous system remains unexplored. This study aimed to characterize the bioactive constituents of GF and evaluate its anti-inflammatory and metabolic regulatory effects in lipopolysaccharide-activated microglia. Phytochemical profiling using LC-HRMS and HPLC identified rutin as a primary bioactive component, present at an exceptionally high concentration (9309 μg/g). In BV-2 microglial and RAW 264.7 cells, GF treatment significantly suppressed the expression of pro-inflammatory cytokines and mediators in a dose-dependent manner. Mechanistic studies revealed that GF specifically modulated the ERK signaling pathway. Furthermore, Seahorse XF analysis demonstrated that GF restored mitochondrial homeostasis by reducing basal respiration and proton leak while significantly enhancing spare respiratory capacity. Finally, conditioned medium from GF-treated microglia improved the viability of N2A neuronal cells. These findings highlight GF as a potent botanical source with significant neuroprotective potential, offering a promising candidate for functional food or nutraceutical applications targeting neuroinflammatory disorders.

## 1. Introduction

*Garrya flavescens S. Wats.* (GF) is a flowering shrub indigenous to diverse habitats, including dry forests, deserts, and shrublands across the southwestern United States and the Mexican regions of Baja California, Sonora, and Chihuahua. Traditionally, GF has been recognized for its gastrointestinal spasmolytic activity [1]; however, its broader pharmacological potential, particularly regarding its neuroprotective effects and underlying molecular mechanisms in the central nervous system (CNS), remains largely unexplored.

Microglia serve as the primary mediators of inflammatory responses within the CNS. When activated by external stimuli—such as pathogens, injury, or ischemia—microglia produce an array of pro-inflammatory cytokines that can exert neurotoxic effects on surrounding neurons and glial cells. Consequently, chronic microglial activation has been heavily implicated in the pathogenesis and progression of various neurodegenerative diseases, including Alzheimer’s disease, Parkinson’s disease, and multiple sclerosis [2,3,4]. Given that neuroinflammation is a hallmark of these neurological disorders, therapeutic strategies specifically targeting microglial hyperactivation have garnered significant attention [5,6].

Lipopolysaccharide (LPS), a potent endotoxin, is a well-established stimulus for inducing inflammatory models both in vivo and in vitro. LPS exposure rapidly triggers microglial activation, leads to synaptic damage, and contributes to long-term cognitive impairments and depressive-like behaviors [7,8]. Therefore, the LPS-induced microglial model provides a robust platform for evaluating the anti-inflammatory potential of natural compounds.

In this study, we aimed to characterize the bioactive components of GF and investigate its potential to suppress LPS-induced neuroinflammation. Using phytochemical profiling, we identified key compounds within GF extracts and demonstrated that GF effectively inhibits the inflammatory response in microglia. Furthermore, we explored the involvement of the extracellular signal-regulated kinase (ERK) signaling pathway in GF’s mechanism of action and assessed its impact on mitochondrial bioenergetics. To our knowledge, this is the first study to provide evidence that GF mitigates the metabolic dysregulation of microglia under inflammatory conditions, suggesting its potential as a novel therapeutic agent for neuroinflammatory disorders.

## 2. Results

### 2.1. Chemical Profiling and Quantitative Analysis of Garrya flavescens Extract

Dried GF leaves were extracted with water or ethanol, and then freeze-dried. For all experiments in this study, the resulting extract powders were freshly mixed in a 1:1 ratio (*w*/*w*) prior to the experiments.

**Figure 1:**
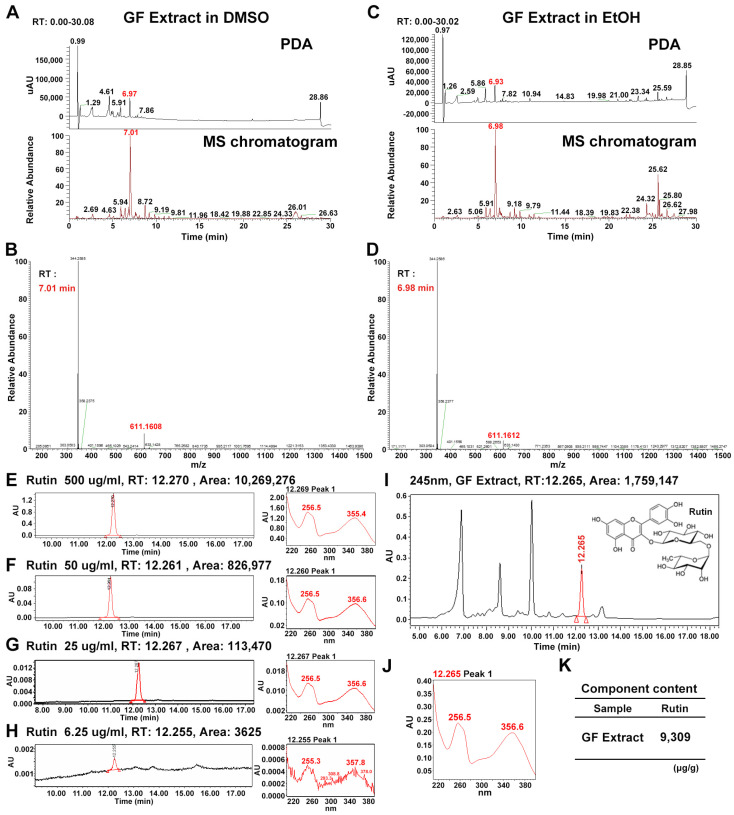
LC-HRMS and HPLC analysis of *Garrya flavescens* extract. (**A**) PDA and MS chromatograms of the GF extract (DW extract: EtOH extract = 1:1) dissolved in DMSO. (**B**) MS spectrum at RT 7.01 min and elemental composition analysis for *m/z* 611.16. (**C**) PDA and MS chromatograms of the GF extract dissolved in EtOH. (**D**) MS spectrum at RT 6.98 min and elemental composition analysis for *m/z* 611.16. (**E**–**H**) HPLC chromatograms of rutin standard at concentrations of 500 (**E**), 50 (**F**), 25 (**G**), and 6.25 (**H**) μg/mL at 254 nm. (**I**,**J**) HPLC chromatograms of the GF extract at 254 nm. (**K**) Rutin content in the GF extract. Abbreviations: GF, *Garrya flavescens*; DW, distilled water; LC-HRMS, liquid chromatography-high resolution mass spectrometry; HPLC, high-performance liquid chromatography; PDA, photodiode array detector; RT, retention time.

To characterize the phytoconstituents of GF, liquid chromatography-high-resolution mass spectrometry (LC-HRMS) was performed on hydroethanolic extracts (50%, *v*/*v*). To ensure the consistency in the profiling, the extracts were analyzed in two different solvents: DMSO (Figure 1A,B) and EtOH (Figure 1C,D). The photodiode array (PDA) and MS chromatogram patterns were highly consistent between the two solvent conditions (Figure 1A,C).

In the DMSO-dissolved extract, major peaks in the PDA chromatogram appeared at retention times (RT) of 5.91, 6.97, 7.86, and 28.86 min (Figure 1A, upper panel). Similarly, the EtOH-dissolved extract exhibited major PDA peaks at RTs of 5.86, 6.93, 7.82, and 28.85 min (Figure 1C, upper panel). The MS chromatograms for both samples also displayed closely matching patterns. Specifically, prominent peaks were observed at RTs of 5.94, 7.01, 9.19, 9.81, 11.96, 18.42, 19.88, 22.85, 24.33, 26.01, and 26.63 min for GF in DMSO (Figure 1A, lower panel), while corresponding peaks in EtOH were identified at 5.91, 6.98, 9.18, 9.79, 11.44, 18.39, 19.83, 22.38, 24.32, 25.80, and 26.62 min (Figure 1C, lower panel).

By comparing the RTs and elemental compositions based on mass-to-charge ratios, a consistently identified peak at *m*/*z* 611.1607 ([M+H]^+^, corresponding to C_27_H_31_O_16_) was tentatively assigned as rutin (Figure 1B,D; Table 1, Table 2 and Table 3).

To confirm the predicted candidates and to analyze the content quantitatively, we performed high-performance liquid chromatography (HPLC) analysis for rutin (Figure 1E–H). In the GF hydroethanolic extract (50% *v*/*v*), rutin was present at an exceptionally high concentration of 9309 μg/g (Figure 1I–K). Rutin standards at concentrations of 500, 50, 25, and 6.25 μg/mL exhibited peaks at RTs of 12.270, 12.261, 12.267, and 12.255 min, respectively (Figure 1E–H, left panels). At these RTs, rutin showed strong absorbance at 256 and 356 nm (Figure 1E–H, right panels). The chromatogram of GF extract also exhibited a peak at RT 12.265 (Figure 1I), where it consistently showed high absorbance at 256.5 and 356.6 nm (Figure 1J), suggesting GF extract includes rutin.

### 2.2. Anti-Inflammatory Activity of Garrya flavescens Extract

**Figure 2:**
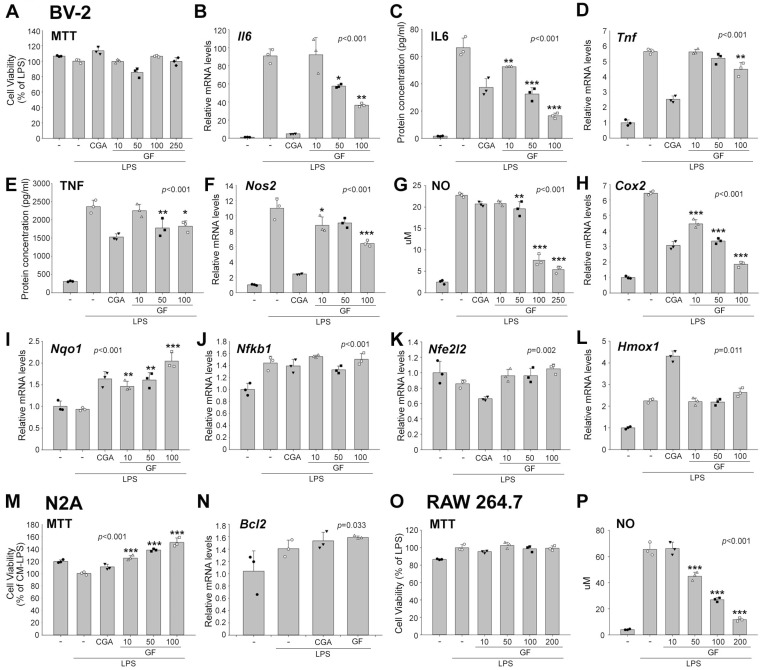
Anti-inflammatory activity of *Garrya flavescens* extract. (**A**–**L**) BV-2 cells were pre-treated with vehicle, chlorogenic acid (CGA; positive control, 4 mM) or GF (10, 50, 100 and 250 μg/mL), followed by stimulation with 100 ng/mL LPS. (**M**,**N**) N2A cells were treated with BV-2-conditioned medium for 24 h. (**O**,**P**) RAW264.7 cells were pre-treated with vehicle or GF (10, 50, 100, and 200 μg/mL), followed by stimulation with 1 μg/mL LPS. (**A**) MTT assay in BV-2 cells. (**B**) Relative *Il6* mRNA levels. (**C**) Supernatant IL6 levels (pg/mL). (**D**) Relative *Tnf* mRNA levels. (**E**) Supernatant TNF levels (pg/mL). (**F**) Relative *Nos2* mRNA levels. (**G**) Supernatant NO levels (μM). (**H**) Relative *Cox2* mRNA levels. (**I**) Relative *Nfkb1* mRNA levels. (**J**) Relative *Nfe2l2* mRNA levels. (**K**) Relative *Nqo1* mRNA levels. (**L**) Relative *Hmox1* mRNA levels. (**M**) MTT assay in N2A cells. (**N**) Relative *Bcl2* mRNA levels. (**O**) MTT assay in RAW264.7 cells. (**P**) Supernatant NO levels (μM) in RAW 264.7 cells. Different groups are indicated by closed and open circles, closed inverted and open triangles, and closed and open squares, respectively. For statistics, one-way ANOVA with the Holm–Sidak post hoc test or Kruskal–Wallis one-way ANOVA on ranks with the Tukey post hoc test were performed. * *p* < 0.05; ** *p* < 0.01; *** *p* < 0.001. N = 3 independent cultures. Bars indicate mean ± SD. Abbreviations: MTT, 3-(4, 5-dimethylthiazolyl-2)-2, 5-diphenyltetrazolium bromide; IL6, interleukin 6; TNF, tumor necrosis factor; NO, nitric oxide; Nos, NO synthase; Cox2, cyclooxygenase2; Nfkb1, nuclear factor kappa B subunit1; Nfe2l2, Nuclear factor erythroid 2-related factor 2; Nqo1, NAD(P)H quinone oxidoreductase 1; Hmox1, heme oxygenase 1; CGA, chlorogenic acid; GF, *Garrya flavescens*; LPS, lipopolysaccharide; Bcl2, B-Cell lymphoma 2; N2A, Neuro-2a.

Rutin is known for its anti-inflammatory activity [9,10]. Since microglia are the major cell type that induce inflammation in the central nervous system [11], we investigated the effects of GF in the microglia cell line BV-2 by using LPS stimulation. Chlorogenic acid (CGA) was included as a positive control due to its established anti-inflammatory properties in microglial models [12] (Figure 2A–L). GF treatment at the concentrations of 10, 50, 100, 250 μg/mL or 4 mM CGA under LPS (100 ng/mL) as well as LPS itself did not impact cell viability (Figure 2A). GF treatment significantly reduced *Il6* mRNA and IL6 protein levels in a concentration-dependent manner (Figure 2B,C) and similarly decreased *Tnf* mRNA and TNF protein levels (Figure 2D,E). *Nos2* mRNA levels, encoding the enzyme that produces nitric oxide (NO), were also significantly reduced by GF (Figure 2F). Consistently, NO production gradually decreased in a concentration-dependent manner upon GF treatment (Figure 2G). The expression of *Cox2*, which facilitates inflammation, was also reduced in a concentration-dependent manner (Figure 2H). GF treatment increased the expression of *Nqo1* (Figure 2I), which reduces LPS-induced production of inflammatory mediators [13], while it did not significantly change the expression of *Nfkb1* (a transcription factor that plays a key role in inflammation), *Nfe2l2* (a transcription factor that has anti-inflammatory properties), or *Hmox1,* which exerts anti-inflammatory effects (Figure 2J–L). This suggests that GF may exert its anti-inflammatory effects through mechanisms distinct from direct modulation of these transcription factors.

To evaluate the potential neuroprotective effects of the GF-treated microglial secretome, we assessed cell viability and the expression of apoptosis-blocking marker *Bcl2* in Neuro-2a (N2A) cells treated with conditioned medium from BV-2 cells that had been treated with GF under LPS-stimulated conditions. GF-treated conditioned medium significantly increased cell viability under LPS stimulation (Figure 2M). However, *Bcl2* expression was not significantly altered by GF treatment (Figure 2N).

In RAW 264.7 cells, GF treatment did not impact cell viability (Figure 2O) and also exhibited a significant, concentration-dependent reduction in NO production (Figure 2P). These findings imply that GF exerts anti-inflammatory functions not only in central nervous system microglia but also in peripheral macrophages.

### 2.3. Signaling Pathways of Garrya flavescens Extract in Microglial Cells Under LPS Stimulation

**Figure 3:**
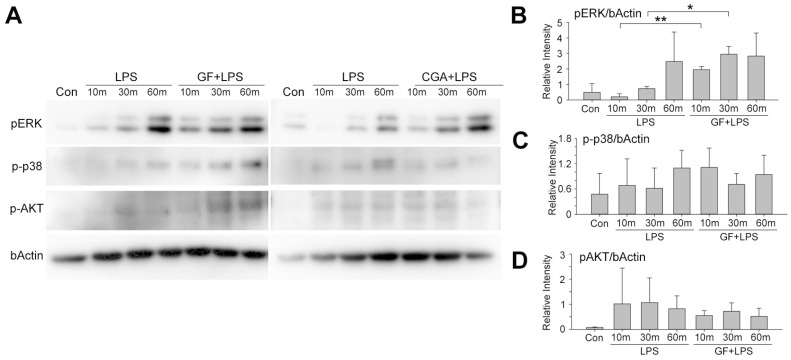
Signaling pathways of *Garrya flavescens* extract under LPS treatment in microglia. (**A**) Western blot analysis of BV-2 cells pre-treated with vehicle or GF, followed by LPS treatment at 10, 30 and 60 min. Antibodies against phosphorylated ERK, p38, AKT and β-Actin were used. (**B**–**D**) Quantitative analysis of the band intensities: p-ERK/β-Actin (**B**), p-p38/β-Actin (**C**), and p-AKT/β-Actin (**D**). * *p* < 0.05; ** *p* < 0.01; Student’s *t*-test. N = 3 independent cultures. Bars indicate mean ± SD. Abbreviations: GF, *Garrya flavescens*; LPS, lipopolysaccharide; CGA, chlorogenic acid; ERK, extracellular signal-regulated kinase; p38, p38 mitogen-activated protein kinase; AKT, protein kinase B; β-actin, beta-actin.

To elucidate how GF signals in microglia under LPS stimulation, we investigated three major signaling pathways: phosphorylation of ERK, p38 and AKT (Figure 3A). Among the three pathways, GF treatment specifically modulated ERK phosphorylation under LPS-stimulated conditions (Figure 3B). The other two pathways were not significantly altered by GF treatment (Figure 3C,D).

### 2.4. Effects of Garrya flavescens Extract on Mitochondrial Function Parameters in Microglial Cells Under LPS Stimulation

**Figure 4:**
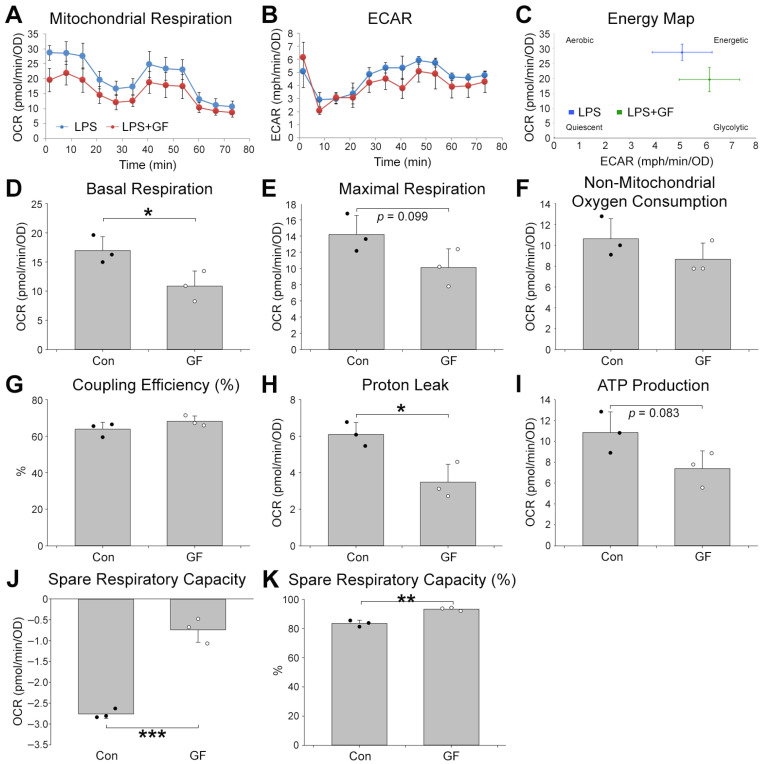
Improvement in mitochondrial function by *Garrya flavescens* extract under LPS treatment in microglia. Seahorse XF cell mito stress test. (**A**) Mitochondrial Respiration. (**B**) Extracellular Acidification Rate. (**C**) Energy Map. (**D**) Basal respiration. (**E**) Maximal respiration. (**F**) Non-mitochondrial oxygen consumption. (**G**) Coupling efficiency (%). (**H**) Proton leak. (**I**) ATP production. (**J**) Spare respiratory capacity. (**K**) Spare respiratory capacity (%) on BV-2 cells pre-treated with vehicle or GF under LPS treatment. Control and GF-treated groups are indicated by closed and open circles, respectively. Student’s *t*-test: * *p* < 0.05; ** *p* < 0.01; *** *p* < 0.001. Abbreviations: GF, *Garrya flavescens*; LPS, lipopolysaccharide.

Mitochondrial dysfunction in microglia is associated with microglia-driven neuroinflammation, a major contributor to the pathogenesis of Alzheimer’s disease [14]. In order to check whether the anti-inflammatory function of GF accompanies the changes in mitochondrial function, we compared mitochondrial parameters in BV-2 cells treated with vehicle or GF under LPS-stimulated conditions using the Seahorse XF system (Figure 4). Oxygen consumption rate (OCR; Figure 4A), extracellular acidification rate (ECAR; Figure 4B), OCR vs. ECAR (Figure 4C) were measured and plotted.

Compared with vehicle, GF treatment significantly reduced basal respiration (Figure 4D) and proton leak (Figure 4H), while it increased spare respiratory capacity (Figure 4J) and spare respiratory capacity (Figure 4K). Other parameters, including maximal respiration (Figure 4E), non-mitochondrial oxygen consumption (Figure 4F), coupling efficiency (Figure 4G), and ATP production (Figure 4I), were not significantly changed.

## 3. Discussion

In this study, rutin, a flavonol glycoside renowned for its potent anti-inflammatory and antioxidant properties, was identified as a primary bioactive constituent of GF. Notably, the rutin content in GF (9309 μg/g) was exceptionally high, surpassing several well-known botanical sources. According to recent comparative studies, this concentration is significantly higher than that of *Thymus serpyllum* L. (875 μg/g), *Artemisia dracunculus* (610 μg/g) and even buckwheat leaves (2700 μg/g) [15,16,17,18,19]. While citrus leaves from orange and lime trees contain comparable levels (11,000 and 7000 μg/g of rutin, respectively) [20], the high density of rutin in GF positions it as a superior ethnomedicinal candidate for neuroprotection, especially considering the ability of rutin metabolites to cross the blood–brain barrier [21,22].

In this study, we employed an LPS-induced inflammation model in microglia. LPS is known to activate microglia and, in vivo, damage GABAergic synapses, leading to long-term cognitive impairments [7]. Suppressing microglial activation has been reported to exert therapeutic effects in depression [8]. Thus, LPS serves as a well-established model for studying neuroinflammation mediated by microglia. The ability of GF to reduce LPS-induced inflammation in microglia suggests that GF administration may exert anti-inflammatory effects not only in vitro in microglia but also in vivo against neuroinflammation, paving the way for future research in this area. Under LPS-induced inflammatory conditions, GF exhibited anti-inflammatory effects in microglia via modulation of ERK phosphorylation (Figure 3). Similarly, several other anti-inflammatory agents mediate their effects through ERK signaling. For instance, schisantherin A activates Nrf2 via ERK phosphorylation, exerting anti-inflammatory and antioxidant effects [23]. Likewise, astragaloside IV activates the NRF2/HO-1 pathway through ERK activation, providing neuroprotective anti-inflammatory effects [24].

LPS-induced inflammation is associated with increased basal ATP consumption and enhanced mitochondrial respiration in microglia, as observed in both this study (Figure 4) and previous research [25]. However, under conditions of high LPS concentration or prolonged exposure, mitochondrial respiration appears to shift toward glycolysis-dominant energy production [25]. GF mitigated inflammation through ERK signaling, thereby reducing the energy consumption required for inflammatory activity. Consequently, GF treatment decreased basal respiration, maximal respiration, and ATP production (Figure 4D,E,I). Proton leak, a phenomenon in which protons leak across the mitochondrial inner membrane, disrupts mitochondrial membrane potential. This process is often induced by LPS exposure and can lead to increased reactive oxygen species production, ultimately causing inflammation and cellular damage [26]. GF reduced proton leak, likely by suppressing inflammation and protecting cells from damage (Figure 4H). Spare respiratory capacity (SRC) represents the additional ATP production capacity of mitochondria in response to increased energy demands. It is essential for cellular survival and function, particularly in processes such as cell proliferation, differentiation, and apoptosis. GF significantly increased SRC under LPS-induced conditions, suggesting that GF enhances cellular adaptability to meet high energy demands in the brain (Figure 4J,K).

In summary, this study provides the first comprehensive characterization of the chemical constituents and pharmacological functions of GF in the context of neuroinflammation. We identified that GF contains exceptionally high levels of rutin, exceeding those reported in many other botanical sources. Functionally, GF effectively suppressed LPS-induced microglial activation and the release of neurotoxic mediators, primarily through modulation of the ERK signaling pathway. In addition, GF restored mitochondrial bioenergetic balance under inflammatory conditions by reducing proton leak and improving spare respiratory capacity. These combined effects suggest that GF exerts both anti-inflammatory and metabolic regulatory actions, supporting its potential as a novel natural candidate for targeting neuroinflammation.

Despite these findings, several limitations should be considered. GF extract comprises multiple phytochemicals beyond rutin, and the overall bioactivity is likely influenced by complex synergistic or antagonistic interactions among these constituents [27]. Therefore, further studies are required to clarify how these compounds collectively contribute to the observed neuroprotective effects. Moreover, as the present study was conducted in vitro, the translational relevance to in vivo systems remains uncertain. In particular, rutin is known to undergo metabolic conversion into derivatives such as quercetin following absorption, which may alter its biological activity [28]. Accordingly, future in vivo investigations are necessary to validate the therapeutic potential of GF and to elucidate its mechanisms of action in neuroinflammation-related disorders.

## 4. Materials and Methods

### 4.1. Plant Material and Extract Preparation

GF leaves were collected in Arizona, USA (Coconino County, 2400 N State Rte 89A, Sedona 86336; 34.887359° N, 111.732555° W; forest near water) and deposited by Hyeran Ihm at the ASU Vascular Plant Herbarium on 4 November 2023 (Specimen No. ASU0160140) (Supplementary Figure S1). Dried GF leaves were ground and sonicated in an equal weight of water or ethanol for two hours at room temperature, followed by freeze-drying. The resulting extracts were freshly mixed in a 1:1 ratio (EtOH extract to water extract) prior to the experiments.

### 4.2. LC-HRMS and HPLC

The chemical profile of the GF extract was analyzed using LC-HRMS and HPLC. Detailed instrument settings and gradient conditions are provided in Table 1 and Table 2, respectively.

### 4.3. Cell Culture and Treatment

The murine microglial BV-2 cell line (derived from neonatal C57BL/6 mouse brain, immortalized with a J2 retrovirus carrying the *v-raf*/*v-myc* oncogenes), the murine macrophage RAW 264.7 cell line (derived from Abelson murine leukemia virus–induced tumor in a male BALB/c mouse), and the murine neuroblastoma N2A cell line (isolated from a mouse brain tumor) were used in this study.

BV-2, N2A, and RAW 264.7 cells were maintained in DMEM supplemented with 10% heat-inactivated FBS and 1% penicillin-streptomycin at 37 °C in a humidified atmosphere containing 5% CO_2_. Cells were confirmed to be mycoplasma-free before use. For inflammation induction, BV-2 cells (500,000 cells/well in 6-well plates; 40,000 cells/well in 96-well plates) were pre-treated with GF, chlorogenic acid (CGA, positive control), or vehicle for 30 min, followed by stimulation with 100 ng/mL LPS. For the neurotoxicity model, N2A cells (800,000 cells/well in 6-well plates) were treated with BV-2-conditioned media for 24 h to assess neuroinflammation-mediated toxicity. For RAW 264.7 cells, treatments were performed under the same conditions as for BV-2 cells, except that the LPS concentration was 1 μg/mL.

### 4.4. Quantitative Real-Time PCR

Total RNA was isolated from dissected mouse brains or cultured cells using TRI Reagent (Sigma-Aldrich, St. Louis, MO, USA) and reverse-transcribed into cDNA using the SuperScript IV VILO Master Mix (Thermo Fisher Scientific, Waltham, MA, USA). Quantitative real-time PCR was performed using PowerUP SYBR Green Master Mix (Life Technologies, Carlsbad, CA, USA). The primer sequences used in this study are as follows. *Il6* (Forward, 5’-TGAACAACGATGATGCACTTG-3’; Reverse, 5’-CTGAAGGACTCTGGCTTTGTC-3’), *Tnf* (Forward, 5’-GGTGCCTATGTCTCAGCCTCTT-3’; Reverse, 5’-GCCATAGAACTGATGAGAGGGAG-3’), *Nos2* (Forward, 5’-GAGACAGGGAAGTCTGAAGCAC-3’; Reverse, 5’-CCAGCAGTAGTTGCTCCTCTTC-3’), *Cox2* (Forward, 5’-GCGACATACTCAAGCAGGAGCA-3’; Reverse, 5’-AGTGGTAACCGCTCAGGTGTTG-3’), *Nqo1* (Forward, 5’-CAGCCAATCAGCGTTCGGTA-3’; Reverse, 5’-CTTCATGGCGTAGTTGAATGATGTC-3’), *Nfkꞵ* (Forward, 5’-GCTGCCAAAGAAGGACACGACA-3’; Reverse, 5’-GGCAGGCTATTGCTCATCACAG-3’), *Nfe2l2* (Forward, 5’-CAGCATAGAGCAGGACATGGAG-3’; Reverse, 5’-GAACAGCGGTAGTATCAGCCAG-3’), *Hmox1* (Forward, 5’-TGCAGGTGATGCTGACAGAGG-3’; Reverse, 5’-GGGATGAGCTAGTGCTGATCTGG-3’), *Bcl2* (Forward, 5’-TGGGATGCCTTTGTGGAACTAT-3’; Reverse, 5’-AGAGACAGCCAGGAGAAATCAAAC-3’).

### 4.5. Elisa, Nitric Oxide Measurement and Cell Viability

Secreted TNF and IL6 levels in the culture supernatants were quantified using Mouse DuoSet ELISA kits (R&D Systems, Minneapolis, MN, USA) according to the manufacturer’s instructions. NO production was determined by mixing equal volumes of conditioned medium and Griess reagent (1% sulfanilamide in 5% phosphoric acid and 0.1% N-1-naphthylethylenediamine dihydrochloride, diluted 1:1 with DW). The absorbance was subsequently measured at 570 nm using a microplate reader. Cell viability was assessed using the EZ-Cytox kit MTT assay (DogenBio, Seoul, Republic of Korea), according to the manufacturer’s instructions.

### 4.6. Western Blot Analysis

Cells were lysed on ice using chilled RIPA buffer (WSE-7420, ATTO, DAWINBIO Inc., Hanam, Korea) and centrifuged at 15,000 rpm for 15 min. The supernatants were collected, quantified, combined with sample buffer, boiled, separated by SDS-PAGE, and transferred onto PVDF membranes. The membranes were blocked with EZBlock Chemi (AE-1475, ATTO, Tokyo, Japan) for 30 min at room temperature and then incubated with primary antibodies overnight at 4 °C. Following washes, the membranes were treated with secondary antibodies for 1 h at room temperature and washed again. Protein signals were visualized using Super Signal West Pico PLUS Chemiluminescent Substrate (34580, Thermo Fisher Scientific, Waltham, MA, USA), and images were captured using the Amersham Imager 600 (GE Healthcare, Chicago, IL, USA). Quantification of the Western blot images was performed with Image J software (version 1.52p; National Institutes of Health, Bethesda, MD, USA).

### 4.7. Antibodies and Chemicals

Rabbit antibodies were purchased from Cell Signaling Technology (Danvers, MA, USA) as follows: phospho-ERK (Thr202/Tyr204, #9101), phospho-p38 (Thr180/Tyr182, #4511T), and phospho-AKT (Thr308, #13038T). Rabbit anti-β-actin antibody (bs-0061R) was obtained from Bioss (Beijing, China). Lipopolysaccharide (LPS; L2630), rutin (PHL89270), and chlorogenic acid (C3878) were purchased from Sigma-Aldrich (St. Louis, MO, USA).

### 4.8. Seahorse Extracellular Flux Analysis

The OCR of BV-2 cells treated with vehicle or GF under LPS was measured using a Seahorse Bioscience Extracellular Flux Analyzer (XF HS Mini; Agilent Technologies, Santa Clara, CA, USA) and the Seahorse XFp Cell Mito Stress Test Kit (Agilent Technologies; Cat. No. 103010-100), following the manufacturer’s protocol. In brief, BV-2 cells were seeded in DMEM at a density of 4000 cells per well, a range optimized through prior cell titration experiments. After several hours, cells were pre-treated with 100 µg/mL GF or vehicle for 1h, followed by 100 ng/mL LPS treatment for 24 h. On the next day, cells were washed with pre-warmed Seahorse assay medium and incubated in a CO_2_-free incubator at 37 °C for 1 h. The stress test was performed by measuring OCR at basal levels and subsequently after sequential injections of oligomycin, FCCP, and rotenone/antimycin-A at final concentrations of 1 μM, 2 μM, and 1 μM, respectively. Data analysis was conducted using the Seahorse XF Cell Mito Stress Test Report Generator (Agilent), which provided values for basal respiration, ATP production, extracellular acidification rate and other related parameters. The data were normalized to protein content per well after OCR measurements.

### 4.9. Statistical Analysis

Data are presented as mean ± standard deviation (SD). Statistical significance was determined using one-way analysis of variance (ANOVA) followed by the Holm–Sidak post hoc test for multiple comparisons. For non-parametric data, the Kruskal–Wallis one-way ANOVA on Ranks followed by Tukey’s test was employed. Comparisons between two groups were performed using Student’s *t*-test. All analyses were conducted using SigmaPlot software (version 14.0; Systat Software, Inc., San Jose, CA, USA). A *p*-values < 0.05 were considered statistically significant (* *p* < 0.05, ** *p* < 0.01, *** *p* < 0.001).

## Figures and Tables

**Table 1:** LC-HRMS Analytical Conditions.

Category	Parameter	Description/Condition
Materials	Solvents	Ultrapure water (Milli-Q); Acetonitrile (Honeywell B&J, Muskegon, MI, USA)
	Reagent	Formic acid (Sigma-Aldrich, St. Louis, MO, USA)
LC System	Instrument	Ultimate 3000 UHPLC system (Thermo Fisher Scientific, Waltham, MA, USA)
	Column	Acquity UPLC BEH C18 (1.7 μm; Waters, Milford, MA, USA)
	Mobile phase	A: 0.1% Formic acid in water; B: 0.1% Formic acid in acetonitrile
	Flow rate	400 μL/min
	Injection volume	2 μL
	Gradient elution	0–1.5 min: 5%B; 1.5–20 min: 70% B
MS System	Instrument	LTQ-Orbitrap XL (Thermo Fisher Scientific)
	Ionization mode	Electrospray ionization (ESI), Positive mode ([M+H]^+^)
	Scan range	PDA: 200–500 nm; MS: *m/z* 150–1500
	Spray voltage	3.5 kV
	Capillary voltage	20 V
	Capillary temp.	350 °C
	Software	Xcalibur (version 4.1; Thermo Fisher Scientific, Waltham, MA, USA)

**Table 2:** Conditions for HPLC.

Parameter	Operating Conditions
Column	Kromasil 100-3.5-C18
Column temperature	Room temperature
Column dimension	4.6 × 250 mm
Flow rate	1 mL/min
Detector	UV Detector
Particle size	5 micron
Detection	Rutin 254 nm
Injection volume	10 μL
Mobile phase condition	Rutin (A. Methanol; B. Water) 0~10 min: 10-65% A; 10~20 min: 65%A; 20~40 min: 65-100%A; 40~45 min: 100%A; 45~47 min: 100-10%A; 47~50 min: 10%A

**Table 3:** Identification of rutin in DMSO and EtOH extracts of *Garrya flavescens* using LC-HRMS. Data were acquired in positive electrospray ionization (ESI^+^) mode. *t_R_*: retention time; Observed *m/z*: experimental mass-to-charge ratio; Theoretical *m/z*: calculated mass based on the formula [C_27_H_31_O_16_]^+^; Δ (ppm): mass error; RDB: ring and double bond equivalents. The identity of Rutin was confirmed by comparing with an authentic standard.

Sample	*t_R_* (min)	Observed *m/z*	Theoretical *m/z*	Δ (ppm)	RDB	Formula ([M+H]^+^)	Identification
GF in DMSO	7.01	611.1608	611.1607	0.28	12.5	C_27_H_31_O_16_	Rutin
GF in EtOH	6.98	611.1615	611.1607	1.37	12.5	C_27_H_31_O_16_	Rutin

## Data Availability

All data generated or analysed during this study are included in this published article and its supplementary information files.

## Supplementary Materials

The following supporting information can be downloaded at: https://www.mdpi.com/article/10.3390/plants15091319/s1, Figure S1: Picture of Garrya flavescens registered in the Arizona State University Vascular Plant Herbarium. Figure S2: Uncropped original Western blot images.

## Abbreviations

ANOVA, analysis of variance; CGA, chlorogenic acid; CNS, central nervous system; ECAR, extracellular acidification rate; ERK (Extracellular Signal-Regulated Kinase): GF, Garrya flavescens S. Wats.; HPLC, high-performance liquid chromatography; LPS, lipopolysaccharide; LC-HRMS, liquid chromatography-high-resolution mass spectrometry; N2A, Neuro-2a; NO, Nitric oxide; OCR, oxygen consumption rate; PDA, photodiode array; RT, retention time; SD, standard deviation; SRC, spare respiratory capacity.
