# Supplementary material for: Anti-Inflammatory Properties of Garrya flavescens: Phytochemical Profiling and Mitigation of LPS-Induced Neuroinflammation via ERK Signaling and Mitochondrial Modulation
Source: Plants (Basel). 2026 Apr 25;15(9):1319. doi: 10.3390/plants15091319 (PMC13165423; doi:10.3390/plants15091319)

Figure S1: Picture of Garrya flavescens registered in the Arizona State University Vascular Plant Herbarium.

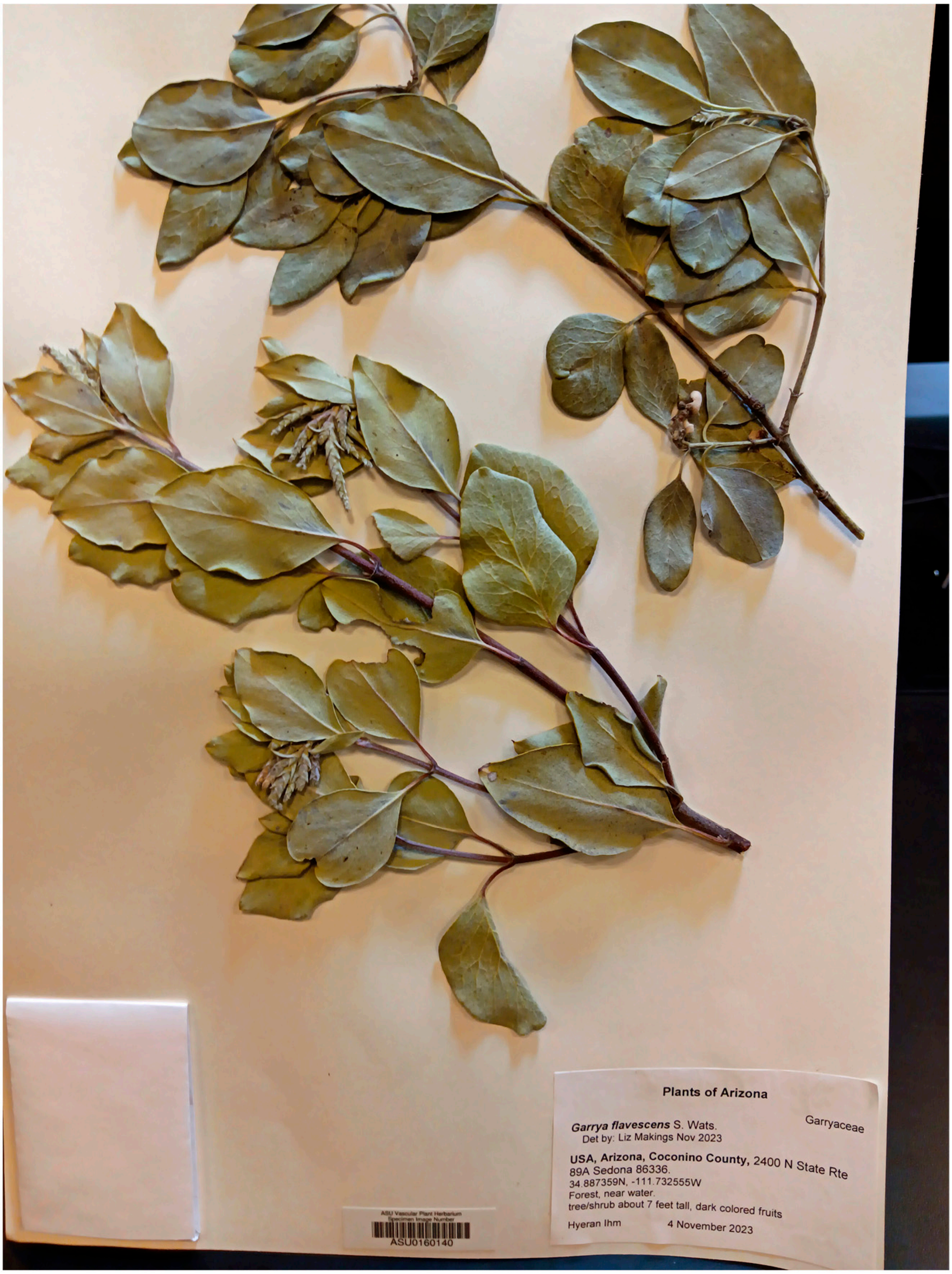

Figure S2: Uncropped original Western blot images.

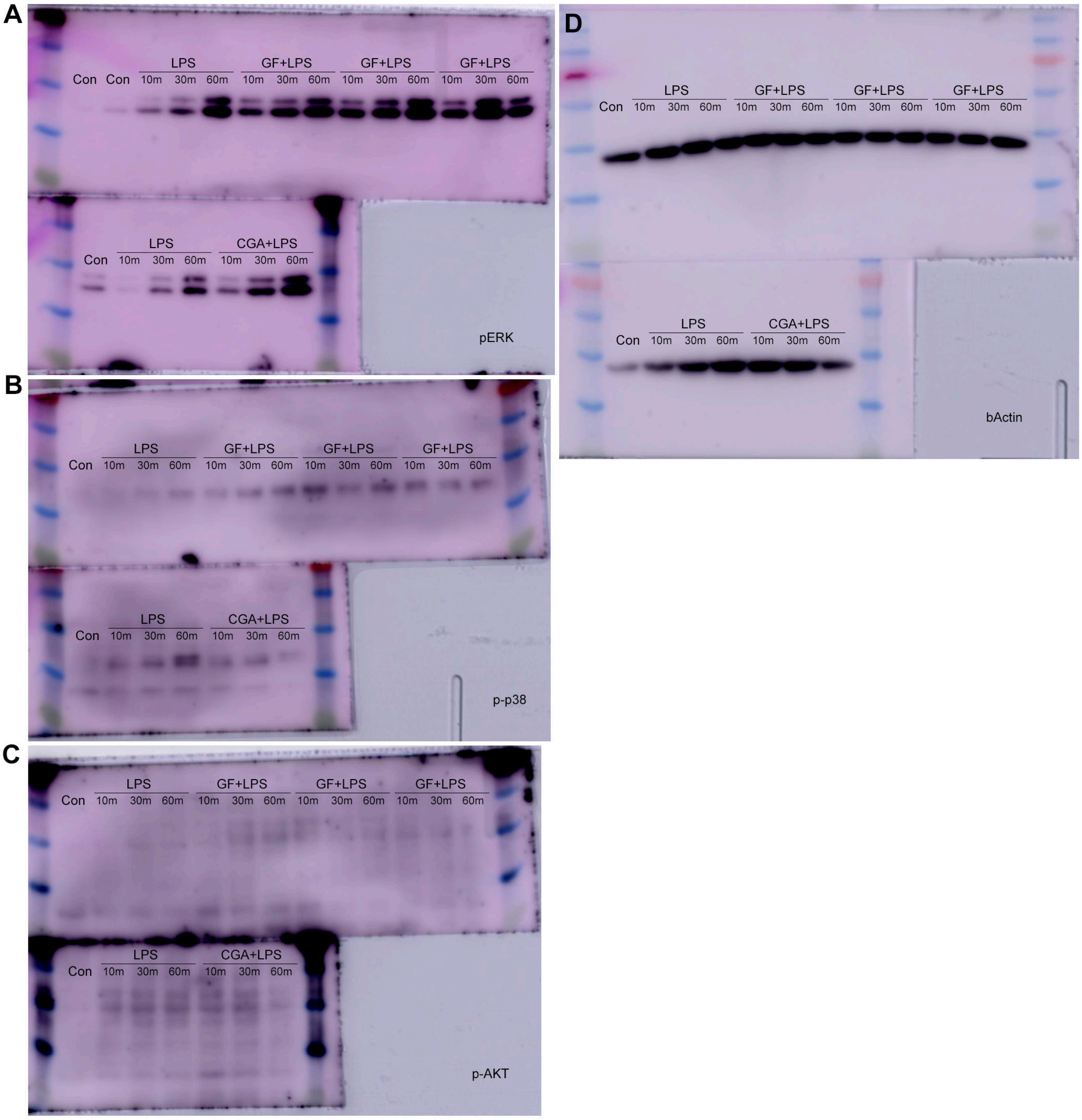

Supplement: Supplementary file 1 [file plants-15-01319-s001.zip › plants-4255275-supplementary.pdf]
